# Medication Safety: Experiential Learning for Pharmacy Students and Staff in a Hospital Setting

**DOI:** 10.3390/pharmacy4040038

**Published:** 2016-11-17

**Authors:** Linda V. Graudins, Michael J. Dooley

**Affiliations:** 1 Pharmacy Department, Alfred Health, Melbourne 3004, Victoria, Australia; m.dooley@alfred.org.au; 2 Centre for Medicine Use and Safety, Monash University, Melbourne 3052, Victoria, Australia

**Keywords:** medication safety, education, pharmacy internship, hospital pharmacy

## Abstract

Medication Safety has been an established pharmacy specialty in Australian hospitals since the early 2000s and is now one of the ten Australian hospital accreditation standards. Although advances have occurred, medication-related patient harm has not been eradicated. Victorian undergraduate pharmacy programs include some aspects of medication safety, however clinical pharmacy experience, along with interpersonal and project management skills, are required to prepare pharmacists to be confident medication safety practitioners. This article outlines the range of medication safety-related training offered at an Australian tertiary teaching hospital, including; on-site tutorial for undergraduate students, experiential placement for pharmacy interns, orientation for pharmacy staff and resources for credentialing pharmacists for extended roles. Improvements continue to be made, such as electronic medication management systems, which increase the safe use of medications and facilitate patient care. Implementation and evaluation of these systems require medication safety expertise. Patients’ engaging in their own care is an acknowledged safety improvement strategy and is enhanced by pharmacist facilitation. Building educator skills and integrating experiential teaching with university curricula should ensure pharmacists have both the knowledge and experience early in their careers, in order to have a leading role in future medication management.

## 1. Introduction

We are into the second decade post the seminal publication of “To Err is Human”, which highlighted error as a significant cause of healthcare-related harm. The review introduced patient safety into healthcare as an important area to be addressed and created a sense of urgency for improvements to be initiated [1]. Improvements have occurred, such as understanding human factors in the context of medical error, incident reporting and evaluation systems and the development of tools to support medication safety initiatives [2,3]. Since 2002, medication safety has been recognized within the Society of Hospital Pharmacists of Australia (SHPA), which created a Community Of Specialty Practice (COSP) and published Standards [4]. The National Prescribing Service (NPS) has also made available a medication safety module, which is mandated in several medical and pharmacy undergraduate courses [5]. 

What is certain from publications, reports, national bodies and local discussions is that patient safety continues to be a concern. For example, a recent review from the United States lists medical error as the third most common cause of death [6]. With regards to Australian data, it is estimated that 2%–3% of hospital admissions are medication-related [7], an incidence unchanged since the initial publication in 2002. The 2013 review lists medication safety improvement strategies implemented in Australia, including the introduction of a standard national medication chart, “smart” infusion pumps and advances in medication reconciliation. In 2012 the Australian Commission for Safety and Quality in Health Care (ACSQHC) included Medication Safety as one of ten health care standards, developed to provide both a quality assurance and quality improvement mechanism for health service organizations [8]. 

Despite research, improvements and awareness, risks to patient safety remain and must be addressed in day-to-day medication management. As professionals in charge of medication management, it follows that pharmacists and pharmacy staff should be trained to detect and prevent errors, as well as be able to provide advice on medication systems to be as safe and effective as possible. 

For the last ten years, medication safety pharmacists at Alfred Health have supervised a pharmacy intern on rotation as part of the pre-registration program and have participated in teaching undergraduate pharmacy students during their experiential placements. For the last five years, a medication safety checklist and face-to-face session has been part of orientation for every staff member in the hospital’s pharmacy department, with the expectation that during their daily work, staff members participate in medication safety initiatives. This paper discusses teaching medication safety for these three approaches to integrate medication safety into pharmacist practice.

## 2. Setting

Alfred Health is an Australian metropolitan, tertiary teaching hospital in Melbourne, Victoria. The Pharmacy Department service covers clinical, procurement and dispensing to patients with a network of three hospitals and over 800 beds. As well as general medicine and surgery, the hospital provides a range of specialist services, including transplantation, burns, infectious diseases, oncology, pediatric, rehabilitation medicine, and psychiatric services. The Pharmacy Department provides a comprehensive seven day a week clinical pharmacy service, with pharmacists attached to each unit-based medical team. There are opportunities to specialize as part of a structured Fellowship program. In-house research projects led by staff pharmacists, under- and post-graduate students are all supported by a research team. Alfred Health employs seven pharmacy interns, whose program is coordinated by a designated pharmacist educator. Each year Pharmacy Department staff supervise over a hundred students from three Victorian Pharmacy schools: La Trobe University, Royal Melbourne Institute of Technology and Monash University. The Pharmacy Department has two senior Medication Safety Pharmacist positions (1.2 Full Time Equivalent), who co-ordinate activities related to Medication Safety across three campuses, including multi-disciplinary Medication Safety and Adverse Drug Reaction (ADR) Review Committees. A regular prescribing newsletter, highlighting incidents, new guidelines or trends, is distributed to nurse managers, pharmacy staff and prescribers. September is designated as medication safety month, “Safetember”, a hospital-wide initiative, involving all campuses and staff in a variety of activities and presentations. 

## 3. Medication Safety Program

### 3.1. Undergraduate Students

Undergraduate students are placed with hospital pharmacists over a two-week period, exposing students to a variety of clinical areas and specialties. Experiential placements aim to provide accelerating learning, help students to reflect and apply learning to new contexts, building on their theoretical knowledge [9,10]. The student handbook outlines the requirements for each area of the placement. Medication safety requirements differ for each university, ranging from descriptive pharmacovigilance, where the student is required to complete an ADR form, to reflecting on broader issues, such as the impact of medication errors on the healthcare system in Australia. Similarly, the university component covering medication safety varies between institutions, ranging from a few lectures on how to undertake medication reconciliation, to a comprehensive week’s worth of medication safety-related topics. During the experiential placement at The Alfred, the medication safety pharmacist holds a didactic tutorial, during which students are encouraged to take opportunities for interaction. A one-hour session covers aspects of medication safety in practice. Students are asked to give examples of “medication safety” from hospital preceptorships, or from experiences gained whilst working in community pharmacy. Resources, such as the Institute for Safe Medication Practices (ISMP) and International Society for Quality in Healthcare (ISQua), are described and the role of national bodies such as the ACSQHC is outlined. A ten-minute film produced for ISMP, “Beyond Blame”, provides an opportunity to discuss actual cases in which medication errors resulted in patient fatalities [11]. Although produced in 1997, the three cases presented, involving a doctor, nurse and pharmacist, are as relevant today as it was when it was filmed 20 years ago. After watching the film, the students are asked to recount each incident, which medications were involved and why the health care worker made the error. They are then asked to outline, if they were the medication safety pharmacist, how they would prevent a similar error from re-occurring. This part of the session provides an opportunity to discuss the effectiveness of various interventions, from applying forcing functions to using education to developing guidelines and how changes are best implemented. Although cases are hospital based, students can reflect on how they may use interventions in both community and hospital pharmacy settings to decrease errors. This session highlights safe use of high risk medications, designated by the “PINCH” acronym: Potassium intravenous, Insulin, Narcotics, Neuromuscular Blocking Agents, Chemotherapy, Heparin and anticoagulants [12]. Cases from published reviews [13] are presented and local medication safety innovations, such as neuromuscular blocking agent management [14] are discussed. Local incidents illustrate how theory can be applied in practice, and highlight how pharmacists can initiate, co-ordinate and implement systems to ensure patient safety. The session places an emphasis on teamwork with nursing and medical staff, along with communication with patients. Students are encouraged to ask their preceptors to show how innovations, such as “smart pump” infusion technology and core practices, such as medication reconciliation, are integrated into daily practice. 

A separate tutorial focuses on adverse drug reactions (ADR) and encourages students to discuss ADRs they have encountered. As students are familiar with ADR terminology and theory, the tutorial emphasizes causality assessment and the importance of accurate ADR documentation. To illustrate medication safety issues, Australian studies around “labeling” of antibiotic allergies [15] and the importance of accurate and clear communication to patients and their health carers [16] are presented.

### 3.2. Pharmacy Interns

To be a registered pharmacist in Australia, pharmacy graduates require a one-year period of supervised training (internship), including oral and written examination. No specific topics or requirements are listed in the Intern Training Program (ITP) standards, but topics and contents must be pertinent to contemporary pharmacy practice [17]. ITP providers must support the intern and through partnership with the preceptor, enable interns to carry out workplace based components of the program. 

At Alfred Health, the intern year is divided into orientation, ten-week rotations in clinical specialties, and two-week rotations in each of Medicines Information and Medication Safety. Throughout the year interns are exposed to opportunities for experiential learning and reflection with weekly tutorials. The medication safety rotation includes a session together with undergraduate students as described above. Each intern participates in the ADR Review Committee meetings. They follow a case from the report, to the patient, preparation for review at the meeting and the written recommendation sent to the patient and documented in medical and pharmacy records [18]. By participating in the complete ADR process, the intern has a better understanding of the importance of reporting, documentation and communication to the patient.

A Drug Use Evaluation (DUE), or quality improvement project, is given to the intern to have an opportunity to learn basic project management, literature searching, data collection, spreadsheets and report writing skills. Intern assignments may be part of scoping for larger projects. One example is the Critical Omitted Dose project, which started as an intern project, and became a multi-centre study [19]. Other intern projects may involve writing a patient information leaflet (PIL) or collecting audit data for the hospital’s Medication Safety Committee. The intern also observes the medication safety pharmacist investigating incidents reported via the hospital’s electronic reporting system. The intern also learns to detect “look-alike sound-alike” (LASA) errors, to produce medication safety alerts for the point of dispensing or administration and the importance of proactive product review [20]. Formal evaluation of the Medication Safety rotation for interns has not been carried out. However, the following statements are examples of subjective feedback from current Alfred Health pharmacists, reflecting on their medication safety intern rotation (year of internship):

I enjoyed my PIL project. I really felt like I had achieved something by producing it. (2015)

… being able to get constant feedback is what I enjoyed the most, having a senior work closely with you to check how you are developing tools or collecting data builds confidence in what you are doing. (2014)

I experienced all components of experiential learning through completion of my project and implementation of strategies to reduce future errors. (2014)

… to work through developing auditing tools, which are important skills required to help me develop an audit for a current project I am involved in. (2012)

… medication adverse drug reaction reports locally and nationally and found attending the ADR meetings really useful. (2011)

… projects, going to the ward auditing patient charts, going to the ADR committee meetings and the other meetings re med safety were the most valuable and are valuable to me still today. (2011)

I saw the results of applied improvements (i.e., new labels on shelves to mark when GTN tablet bottles were opened). I have the confidence to complete an ADR form and attend meetings. (2010)

### 3.3. Pharmacy Staff

Each staff member (intern, technician, pharmacist, procurement staff, and researcher) undergoes Pharmacy Department orientation in the first weeks of employment. Orientation with the Medication Safety pharmacist involves a face-to-face session, either one-on-one or small group. The session includes; medication safety resources available on the intranet, reporting a medication-related incident on-line, reporting an ADR, optimal use of systems, such as bar code scanning and insulin dose validation. 

Medication safety pharmacists present at pharmacy educational sessions, as well as being involved in staff reflective sessions, where pharmacy-related incidents are discussed. A regular section in the pharmacy newsletter facilitates Medication Safety as part of everyday practice.

An on-line safe prescribing module was developed in 2011 by the medication safety pharmacist, with the hospital’s Post Graduate Medicine coordinator. Medical interns must now complete the module and attend the face-to-face session at orientation to become credentialed to prescribe [21]. With the increasing demand on acute care services, together with a focus on timely completion of care, partnered pharmacist charting on patient admission is being adopted, preventing and reducing drug-related problems. The safe prescribing module is now used as part of pharmacist credentialing for advanced practice roles, whenever pharmacists chart patients’ regular medications [22].

## 4. Pharmacy Based Medication Safety Programs Preparing for the Future

Medication safety pharmacists will need to be prepared for the changes occurring in electronic medication management (eMM) systems and be able to facilitate patients to safely manage their own medicines using technological solutions. These advances require educators to provide opportunities to up-skill current pharmacists and integrate these aspects of medication safety into curricula.

Electronic medication management systems have been available for decades and their use with decision support, has been shown to reduce medication errors. Medication safety experts have seen eMM as a solution to medication safety problems for the 21st century [11]. However, the variety of eMM systems available attest to the fact that after two decades, there is not yet a perfect solution [23]. If electronic medication management is integrated effectively within existing systems, optimal prescribing, documentation and communication should occur, leading to seamless transitions between hospital and the home. Pharmacists with medication safety expertise, along with medical, nursing and technical experts, will need to be involved in the evaluation of these systems, ensuring safety features are optimised and new errors are intercepted. In the coming years, implementation of e-health will be a major focus of medication management and should be a part of pharmacy curricula. 

An important trend in healthcare is the shifting emphasis to the patient being central to medications management. In recognition of this change, “Partnering with Consumers” is an ACHS healthcare accreditation standard [24]. Electronic solutions are replacing traditional paper-based medication lists and consumer information materials and provide opportunities for more effective communication on safe and effective use of medicines throughout the patient’s healthcare experience. However, to be successful, communication must be in a format that patients want and understand. Attempts at a universal electronic health record have not been successful in Australia. Uptake of the national Personally Controlled Electronic Health Record (now MyHealth) launched in 2012, has been poor; by July 2016, only 16% of Australia’s population was registered for the scheme [25]. To address this gap and ensure safe and effective medication use, pharmacists must proactively engage patients to assist in their own medication management. The marketing of pharmacy services via media will need to be adapted into and adopted by health care systems and patients. For example, by using personal electronic devices, which are now commonplace. In outpatient settings, pharmacists can facilitate the use of applications, such as the National Prescribing Service application, for patients to store their medication information [26]. Because of the increasing complexity of medication management and an ageing population with multiple co-morbidities, pharmacists in both hospital and general practice clinics, and in outreach services, are extending their practices and providing more tailored patient-centred care. 

Pharmacists will need to be up-skilled to enable mentoring and teaching of the next generation of students and interns. Skills include being able to apply health-related technologies, such as using social media. Introducing teaching and mentoring into daily practice is required, for example the “Teaching on the run” program [27]. Integration of the hospital placement and intern program with university curricula, along with sharing of knowledge across universities and hospitals will enable pharmacists to be more effective preceptors. Pharmacy schools and professional organisations should be actively involved in advancing Medication Safety in their curricula, ensuring a consistently high standard for students and pharmacists. 

Both current and new educational program should be evaluated to ensure content, structure and outcomes are meeting program aims.

## 5. Conclusions

Medication safety in Australia is a recognized specialty of pharmacy practice. In Victoria, it is integrated into undergraduate curricula and is part of experiential intern training. At Alfred Health, pharmacy intern rotation, staff orientation and tutorials for undergraduate students attest to the importance of education in medication safety. A safe prescribing module is used to credential medical interns and to support extended roles for pharmacists. There have been huge advances in medication safety over the last decade leading to staff awareness, systems improvements and promotion of medication safety via national accreditation standards. Pharmacist skills are evolving along with research in this specialty, to further improve safety of medication management for patients and healthcare staff. 

Electronic medication management is being implemented more widely in Australia, but how to do so effectively and safely is still an area for discussion. The best way for patients to use technology for engaging in their own health care needs to be elucidated. Closer integration of university curricula and hospital practice to inform and improve medication safety will enable pharmacists to continue to contribute to effective and safe patient care.
